# Rat Calvaria Model Mimicking the Intraoral Lesion of Medication-Related Osteonecrosis in the Jaw: A Preliminary Test

**DOI:** 10.3390/jcm12216731

**Published:** 2023-10-25

**Authors:** Yesel Kim, Jeong-Kui Ku

**Affiliations:** 1 Department of Dental Hygiene, Jeonju Kijeon College, Jeonju 54989, Republic of Korea; 2 Department of Oral and Maxillofacial Surgery, School of Dentistry and Institute of Oral Bioscience, Research Institute of Clinical Medicine of Jeonbuk National University, Biomedical Research Institute of Jeonbuk National University Hospital, Jeonbuk National University, Jeonju 54907, Republic of Korea

**Keywords:** dentistry, rat model, osteonecrosis, bisphosphonate-associated osteonecrosis of the jaw, wound healing

## Abstract

Numerous preclinical intraoral models have been proposed to study medication-related osteonecrosis of the jaws (MRONJ). However, an extraoral animal model is necessary to investigate the effects of interventions such as grafts or direct therapeutics. This study aimed to establish a MRONJ rat model on the calvaria. Seven rats were allocated to either the control or MRONJ group. The MRONJ group received injections of zoledronic acid and dexamethasone to induce osteonecrosis over 4 weeks. Two weeks after these injections, the maxillary first molar was extracted, and two calvaria defects were created using a 4 mm trephine burr. One defect was left untreated, while the other was filled with harvested calvaria bone. A histological examination of all calvaria in the MRONJ group revealed avascular necrosis and the destruction of cortical bone. An independent *t*-test and Pearson’s correlation coefficient were used for statistical analysis and the evaluation of alveolar and calvaria defects. The total alveolar and calvaria defect volume in the control group was significantly smaller than that in the MRONJ group. A statistically significant correlation was observed between alveolar and calvaria defects (Pearson correlation = 0.6, *p* = 0.023). The autogenous grafts showed poor results in the MRONJ group since they failed to revascularize and exhibited necrosis. The calvaria in this study successfully mimicked MRONJ lesions with avascular necrosis. This preclinical model could be used to develop treatments that are applicable to MRONJ.

## 1. Introduction

Bisphosphonates have been applied to treat hypercalcemia of malignancy, Paget’s disease, and osteolytic lesions of multiple myeloma [1]. Additionally, they have shown excellent results in preventing and treating osteoporosis. Bisphosphonate-related osteonecrosis of the jaw (BRONJ) is a well-known complication of orthopedic surgery involving bisphosphonates in the oral and maxillofacial regions and atypical femoral fracture [2]. The American Association of Oral and Maxillofacial Surgeons (AAOMS) published a position paper in 2014, the third edition, providing an extended definition of BRONJ as medication-related osteonecrosis of the jaw (MRONJ): current or previous treatment with antiresorptive or antiangiogenic agents, exposed bone in the maxillofacial region for more than eight weeks, and no history of radiation therapy to the jaw bone [3].

Although MRONJ is relatively rare, it has a significant impact on health. Symptoms include delayed wound healing, the loss of oral mucosa, continued pain from exposure, necrosis, and purulent secretion from the jaw bones. Since its initial report in 2003, MRONJ has been reported globally with an incidence ranging from 0.01% to 18.6% [4,5,6,7,8]. The risk of developing MRONJ may increase with the prolonged use of bisphosphonates beyond three years. A drug holiday of approximately one year after five years of drug therapy has been recommended [9]. Despite significant research on MRONJ, its pathogenesis, pathophysiology, and treatment protocols have not been fully elucidated [10,11]. 

Animal research, using rats, mice, rabbits, dogs, and pigs, has contributed immensely to the advancement of the study of MRONJ. Rodent models exhibit excellent effectiveness and high reproducibility at mimicking human MRONJ, using drug administration (zolendronate as antiresorptive drugs or denosumab as anti-receptor activators of NF-κB ligand antibody, plus dexamethasone), mechanical stimuli (bone drilling, tooth extraction, implantation, and occlusal overload), and infection (pulpitis and periodontitis)-inducing methods [3,12,13,14,15,16]. Gross MRONJ presents with unhealed mucosa and exposed bone observable to the naked eye. The most important hallmark of MRONJ is the necrotic bone, which is identified as an area of at least 5–10 confluent empty or karyolytic osteocyte lacunae. The area of necrotic bone was defined as the bone area with empty osteocyte lacunae [17]. Micro-computed tomography (µCT) imaging is the most commonly used method [12]. Currently, the most versatile MRONJ model combines systemic drug injection with healthy tooth extraction in rats because of their rapid reproduction, easy maintenance conditions, and low cost [12,18]. While several reproducible intraoral rat models have been proposed to mimic human MRONJ lesions, implementing interventions for clinical use, such as bone graft or direct therapeutics in the oral cavity, remains exceptionally difficult in these animal models. 

Because calvarial bone defects have been extensively used to evaluate bone tissue response and regeneration, MRONJ-induced calvaria defects could greatly contribute to the development and establishment of MRONJ treatment. Several studies have reported the possibility of MRONJ induction in the skull bone. Choi et al. demonstrated that bisphosphonate (pamidronate) inhibits bone healing in rabbit calvarial bony defects [19]. In 2016, calvarial bone osteonecrosis was reported in a patient treated with bisphosphonates [20]. In 2018, a review article reported that six patients taking bisphosphonates that showed ear canal osteonecrosis [21]. In 2021, True et al. first reported bilateral osteonecrosis of the external auditory canal was associated with denosumab and bisphosphonates [22]. Thus, we hypothesize that MRONJ lesions could also be induced in the calvaria since the jaw, except for the condylar process, and calvaria are originally formed from the same intramembranous bone ossification. This study aims to establish an MRONJ rat model on the calvaria, employing clinical, radiological, and histological measures to demonstrate the correlation between volumetric bone healing in the alveolar and calvaria defects through µCT.

## 2. Materials and Methods

All animal procedures strictly followed the Jeonbuk National University Hospital Institutional Animal Care and Use Committee guidelines for the care and use of laboratory animals (JBUH-IACUC-2023-12) and ethical principles for animal experimentation established by the institute. The sample size was initially calculated as 14 animals using two experimental groups, considering a significance level of 5% and a statistical test power of 95%. To rationalize and distribute the animals, we considered seven animals for each control and experimental group as suitable for the confirmation and evaluation of the MRONJ model and for sacrifice during extraction and calvaria surgery.

### 2.1. Experimental Protocol

**Figure 1:**
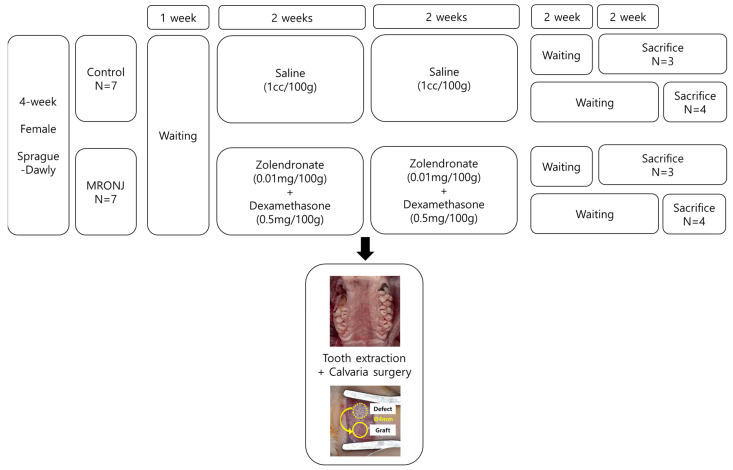
Experimental design. Fourteen 4-week-old Sprague–Dawly rats were allocated into two groups. Tooth extraction and calvaria surgery were performed at two weeks of injection using saline for the control group and zolendronate and dexamethasone for the MRONJ group. The rats were injected for an additional two weeks after surgery. Three and four rats from each group were sacrificed on the fourth and sixth weeks after surgery.

Four-week-old female Spraque–Dawley rats were purchased from Koatech (Pyeongtaek, Kyungki-do, Republic of Korea). Rats were housed at 22 ± 2 °C with a 12 h LD cycle under 55–60% humidity in the SPF facility. Animals were provided with a standard chow diet with free access to water. Fourteen rats were divided into two groups: a control group (*n* = 7) and an MRONJ group (*n* = 7). All rats in the control groups received injections of normal saline (1 cc/100 g), while rats in the MRONJ group were injected with zoledronic acid (0.01 mg/100 g) and dexamethasone (0.5 mg/100 g). These injections were administered three times per week for four weeks (Figure 1).

**Figure 2:**
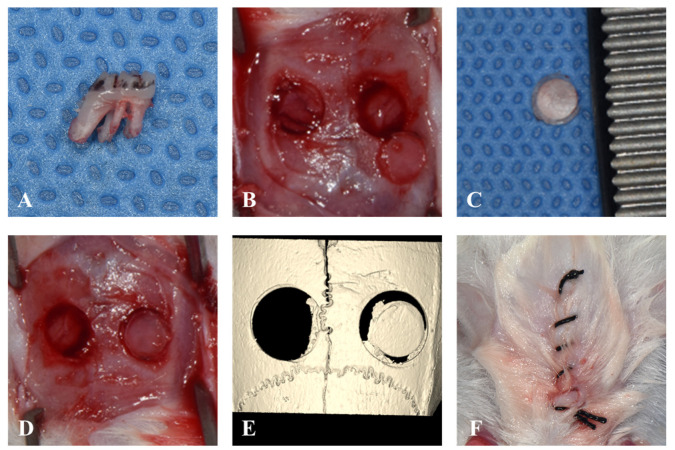
Surgical procedure. (**A**). Extraction of maxillary first molar without any root fractures. (**B**). Two defects in the calvaria were created by A 4 mm trephine burr. (**C**). Harvested autogenous bone from the calvaria. (**D**). The autogenous bone was grafted in the right calvaria defect. (**E**). μ-CT images taken immediately after the surgery. (**F**). The suture line located on the mid-sagittal area to avoid the surgical area.

Two weeks after injection, the maxillary first molar was extracted using a dental explorer, and two calvaria defects were created using a 4 mm trephine burr in all rats under general anesthesia (2.2% isoflurane and oxygen 2 L/min). All surgical procedures were performed under general anesthesia induced with zolazepam (Zoletil 50; Virbac Carros, France) and xylazine hydrochloride (Rompun; Bayer Korea, Seoul, Republic of Korea). One of the two created calvaria defects was left empty, while the other side was grafted with a 4 mm circularly harvested autogenous bone block taken from the opposite side. Primary closure was performed along the mid-sagittal line to avoid suturing above the surgical area (Figure 2). 

### 2.2. Evaluation Methods

**Figure 3:**
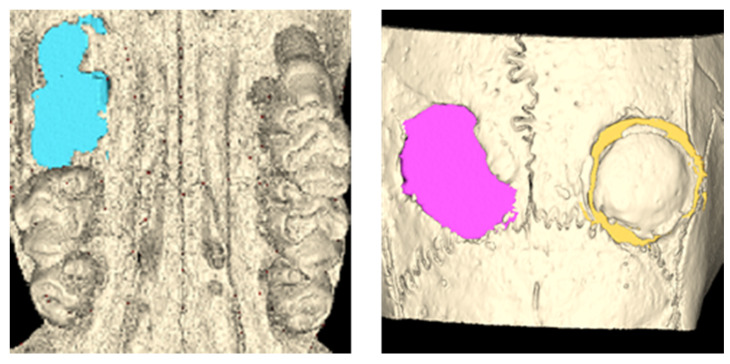
Volumetric measurement using μ-CT. After a ROI was delineated according to the defect, the border of the defects was automatically generated for each slice. The defect volume was calculated through 3D reconstruction.

In each control and experimental group, three rats were sacrificed four weeks after surgery, while the remaining four rats were sacrificed six weeks after surgery. After sacrifice, the maxilla, including the calvaria, was carefully excised and examined to assess bone- and soft-tissue healing within the defect area. All samples were analyzed using μ-CT at the Center for University-Wide Research Facilities (CURF) at Jeonbuk National University (Jeonju-si, Republic of Korea). The samples were evaluated using a SkyScan 1076 (Bruker, Karlsruhe, Belgium) with a pixel size of 35 µm. The following parameters for the CT scanner were set: 100 kV voltage for the X-ray tube, 100 μA current for the X-ray source, and 190 ms of exposure time. The detector and X-ray source were rotated by 0.6° in steps of 360°. The scanned images were reconstructed using NRecon software (Bruker, Karlsruhe, Germany). CT scan projection images and reconstructions were saved as 16-bit TIFF files. These image files were then transferred to Materialise’s interactive medical image control system (MIMICS Version 25.0, Materialise, Leuven, Belgium), which was used to measure the volume [23]. The CT images were converted into Digital Imaging and Communications in Medicine (DICOM) files and imported into MIMICS. The threshold value (1500–3071 Hounsfield units for bone demonstration) was reconstructed for the measurement [24,25]. Once a region of interest (ROI) was automatically delineated in each sample, one examiner (J.K.K.) confirmed the border of the alveolar and calvaria residual defects on the axial and sagittal views, respectively. The defect volume was automatically generated via 3D reconstruction [26] (Figure 3).

The samples were then fixed with a 4% (*w*/*v*) paraformaldehyde solution and stored for five days. After μ-CT scan acquisition, decalcification was carried out using a 10% ethylenediaminetetraacetic acid (EDTA) solution for one week at room temperature. The treated samples were dehydrated, embedded in paraffin, and then cut into 4 µm-thick sections. Stained tissue slides were examined using an Olympus BX51 microscope (Olympus, Tokyo, Japan). Digital images of the selected sections were captured using a digital camera (DP-73; Olympus, Tokyo, Japan).

The Shapiro–Wilk test was used to analyze parametric data for the normality of data. An independent *t*-test was used to compare the groups. The correlation between the alveolar and calvaria defect was analyzed using Pearson’s correlation coefficient. Analyses were performed using IBM SPSS Statistics (version 27, SPSS Inc., Chicago, IL, USA), and differences were considered significant at *p* < 0.05.

## 3. Results

### 3.1. Alveolar Defect

**Figure 4:**
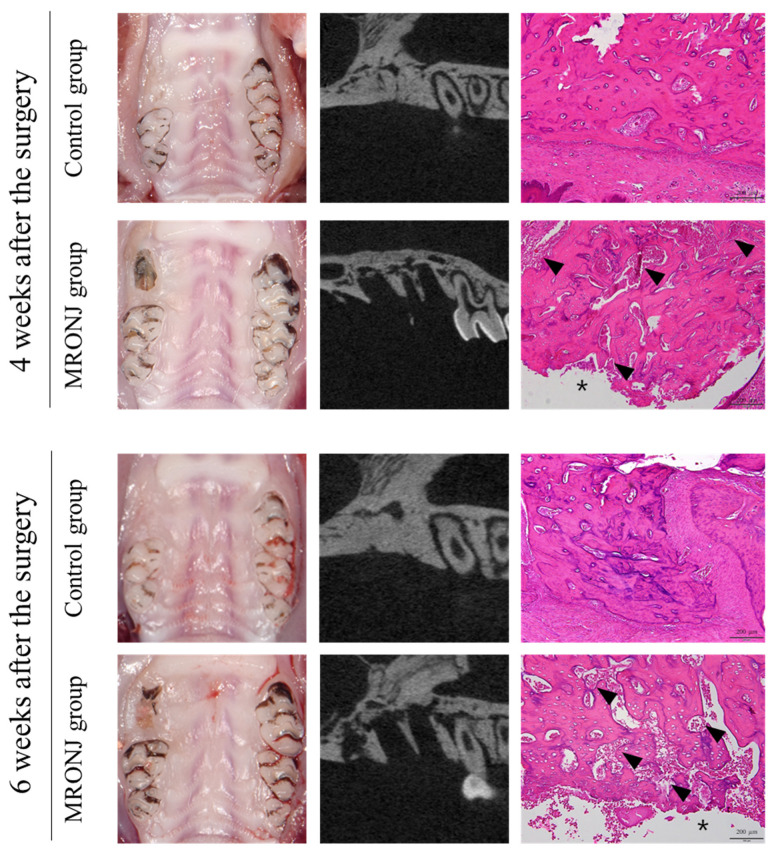
Extraction socket at postoperative 4 and 6 weeks. The control groups achieved gingival and alveolar bone healing after four weeks, while the MRONJ groups revealed bone exposure without alveolar bone healing. The μ-CT of the MRONJ groups showed the sequestration of alveolar bone, and histologic images demonstrated a large percentage of empty osteocyte lacunae with the destruction of the bone matrix (arrowhead) and no epithelial regeneration (asterisk).

**Figure 5:**
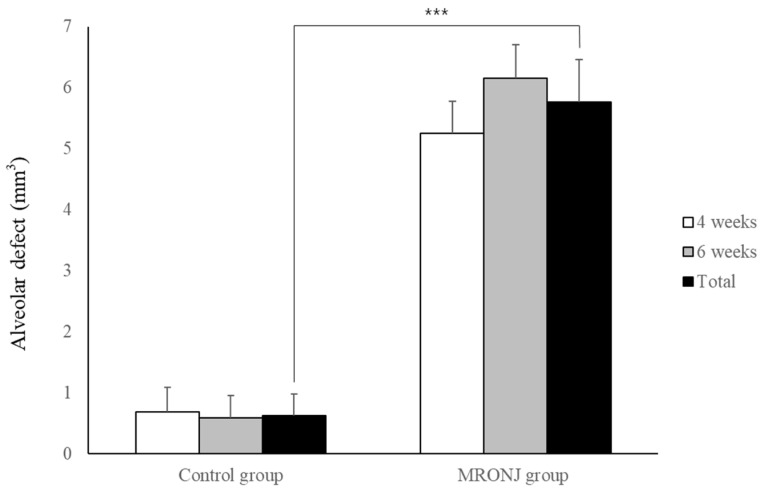
Volumetric analysis of alveolar defect. Each group showed similar results between two time points. The alveolar defect of the control group was significantly reduced compared to the MRONJ groups. (*** *p* < 0.001).

All rats in the control group showed complete gingival healing of the socket, whereas all rats in the MRONJ group showed incomplete gingival healing and exposed necrotic bone. (Figure 4) In μ-CT, the volume of the alveolar defect was significantly reduced in the control groups (0.68 ± 0.40 and 0.58 ± 0.37 mm^3^ at two and four weeks) compared to that of the MRONJ groups (5.25 ± 0.52 and 6.15 ± 0.56 mm^3^ at four and six weeks, Figure 5) The volume of alveolar defects was similar between two time points in each group, and the average alveolar defect in the control group was smaller than the MRONJ group (*p* < 0.001, Figure 5).

### 3.2. Calvaria Defect

**Figure 6:**
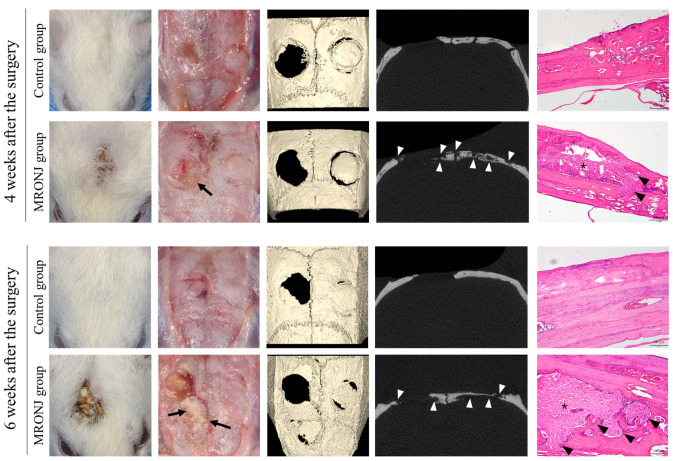
Calvaria at postoperative 4 and 6 weeks. The control groups achieved intact soft tissue healing, while the MRONJ groups revealed incomplete skin healing with dehiscence and necrotic bones (black arrow). The μ-CT of the MRONJ groups showed the sequestration of calvaria bone on both the defect and autogenous bone graft areas (white arrowhead), while histologic images demonstrated a large percentage of empty osteocyte lacunae with the destruction of the bone matrix (arrowhead) and chronic inflammation (asterisk).

In the control group, all rats showed normal healing of the calvaria skin, whereas five out of the seven rats in the MRONJ group showed skin dehiscence and exposure to the calvaria. All rats in the MRONJ group had necrosis of the calvaria (Figure 6).

**Figure 7:**
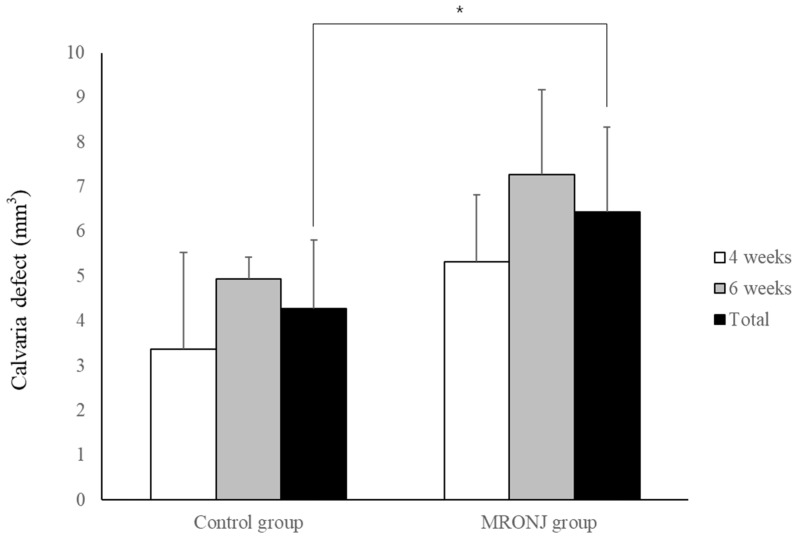
Volumetric analysis of calvaria defect. Each group showed similar results between two time points. The calvaria defect of the control group was significantly smaller compared with the MRONJ groups. (* *p* < 0.05).

In μ-CT, the volume of calvaria defect in the control groups was 3.37 ± 2.16 and 4.93 ± 0.48 mm^3^ at four and six weeks, and 5.33 ± 1.50 and 7.28 ± 1.89 mm^3^ in the MRONJ groups at four and six weeks, respectively. The volume of calvaria defects was similar between two time points in each group without statistical significance, and the average calvaria defect in the control group (4.26 ± 1.54 mm^3^) was smaller than the MRONJ group (6.44 ± 1.90 mm^3^) (*p* = 0.036, Figure 7).

**Figure 8:**
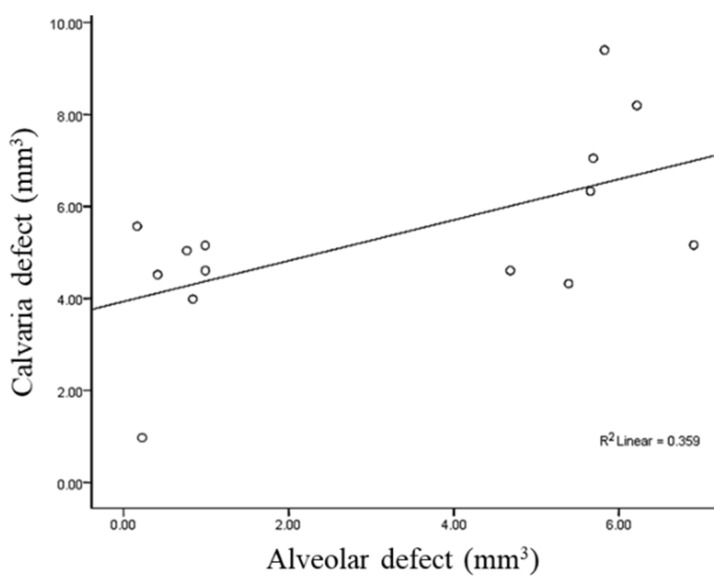
Measure of Pearson’s correlation coefficient to evaluate the relationship between the volume of alveolar and calvaria defects.

The Pearson’s correlation coefficient for the volume between the alveolar and calvaria defect was 0.60 with statistical significance (*p* = 0.023, Figure 8).

### 3.3. Autogenous Bone Grafts in Calvaria Defect

**Figure 9:**
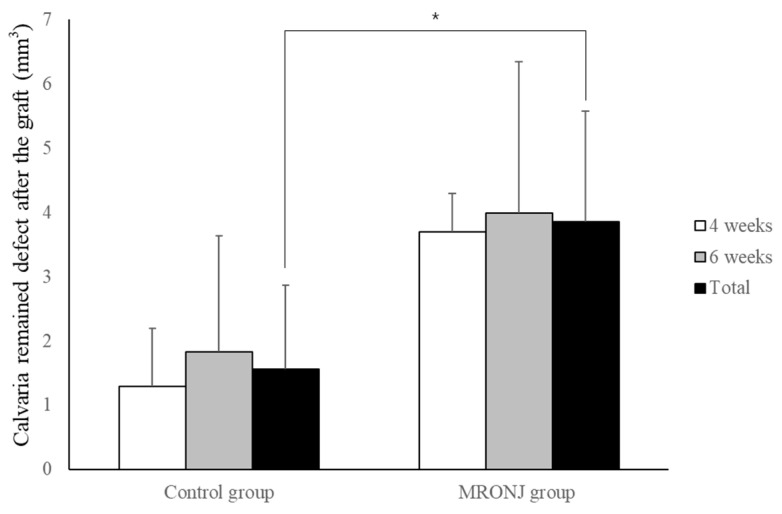
Volumetric analysis of remaining calvaria defects after autogenous calvaria bone graft. Each group showed similar results between two time points. The remaining defect of the control group was significantly smaller compared with the MRONJ groups. (* *p* < 0.05).

With regard to autogenous bone grafts, grafted autogenous bone was adequately healed in the control groups. However, grafts in the MRONJ group showed the osteophytic change and destruction of the bone matrix. The remaining volume in the control groups (1.29 ± 0.90 and 1.83 ± 1.81 mm^3^ at four and six weeks) was smaller compared to the MRONJ groups (3.69 ± 0.60 and 3.99 ± 2.36 mm^3^ at four and six weeks. The remaining volume was similar between the two time points in each group, and the average remaining volume in the control group (1.56 ± 1.31 mm^3^) was smaller than that in the MRONJ group (3.86 ± 1.71 mm^3^) (*p* = 0.021, Figure 9).

## 4. Discussion

The authors assumed that similar to mechanical trauma, such as tooth extraction in an MRONJ rat model, which can resemble human MRONJ lesions, calvaria ostectomy could also induce lesions resembling human MRONJ. In this study, necrosis was induced in all MRONJ rats, and calvaria exposure was observed in 71.4%, although primary closure was achieved in all animals. Furthermore, since there was a volumetric correlation between alveolar and calvaria defects, this model could be available and serve as a rationale for MRONJ treatments such as bone grafting, rhBMP-2, or stem cell applications to the calvaria instead of intraoral surgery.

This calvaria model is suitable for the evaluation of complex materials and tissue-engineered constructs that are aimed at regenerating craniofacial bone defects. This is because the calvarial bone structure allows the creation of a uniform, reproducible, and standardized defect [27,28,29]. The anatomical location also provides an adequate size for surgical access and intraoperative handling. The dura and overlying skin provide adequate support for the implanted materials without internal or external fixation [27]. Bosch et al. reported that a 5 mm defect could not heal spontaneously for up to 12 months after surgery. The advantages of using a 5 mm defect include the possibility of creating two defects per animal and avoiding the inclusion of a sagittal suture in the defect [30]. In our study, the autogenous bone obtained with a trephine burr was 4 mm thick, but due to the thickness of the burr blade, a 5 mm calvaria defect was created. Complete bone recovery was not observed in the control group even after six weeks (Figure 7).

Although some studies have reported intraoral intervention in MRONJ rat models [31,32], there are several differences between rats and humans in terms of the oral environment. Firstly, diets differ between humans and rats, and the composition of oral resident flora may differ. The accessibility of intraoral surgery in rats is poor, and positioning graft materials or local therapies in small oral spaces is challenging. Achieving primary closure via flap management remains challenging [33]. Therefore, most animal experiments on MRONJ have focus on systemic interventions [34]. Since many patients with MRONJ require surgical treatment, we expect that the MRONJ-like calvaria defect presented in this study will provide valuable scientific evidence. Autologous bone is generally considered the gold standard for bone grafting. In 2016, 15 patients taking bisphosphonates for osteoporosis were successfully treated with extensive autogenous bone grafting procedures [35]. However, in cases where MRONJ has already occurred, there is a concern that the lesion has avascular necrosis, and the harvested autologous bone may not integrate without necrosis. According to our results, avascular necrosis was observed in the defect areas. Even around the defects created for bone harvesting and grafting, bone necrosis and destruction were more pronounced. (Figure 6) Furthermore, the remaining bone defect volume after bone grafting also showed statistically significant differences in the MRONJ group on the μ-CT (Figure 9). However, even when measured as a bone area on μ-CT, many areas appeared histologically necrotic in the MRONJ groups. Therefore, autogenous bone alone is unlikely to yield successful results in the treatment of MRONJ defects. The use of rat calvaria has been extensive in studying different bone-healing effects [36,37,38]. Therefore, this rat model for the medication-related osteonecrosis of the jaw (MRONJ) can contribute to research on direct interventions and treatment options for MRONJ therapy. Even in the absence of bacterial infection, the progression of avascular necrosis suggests that further research is needed, considering the angiogenic effects and osteoinductive materials such as rhBMP-2, demineralized dentin matrix, and stem cell therapy. 

This study has several limitations. First, statistical analysis could not be performed for the effect of the bone graft on the calvarial defect in the MRONJ group due to insufficient animals and other experimental groups. This is considered a limitation because the confirmation of the occurrence of MRONJ in the calvaria was conducted as a preliminary test. Second, it is necessary to gather more evidence on whether calvarial bone healing and necrosis progression patterns accurately mimic the physiobiology of the human jawbone in the area where MRONJ occurs. Third, it is challenging to reproduce bacterial infections that play a key role in the development of MRONJ symptoms in humans. 

## 5. Conclusions

This study established a rat model of MRONJ in the calvaria, providing a valuable animal model for understanding and managing MRONJ. This model mimics oral MRONJ lesions and demonstrates a significant correlation between the volume of calvaria defects and intraoral defects. 

## Data Availability

The datasets used and/or analyzed during the current study are available from the corresponding author on reasonable request.
